# Determinants of participation and support mechanisms in a paralympic sport training program: evidence from the IDRD program in Bogotá

**DOI:** 10.3389/fspor.2026.1882854

**Published:** 2026-07-03

**Authors:** Stevens Ruiz, Mireille Jasmin, Natalia Varela Pulido, Mauricio Garzon, Tegwen Gadais

**Affiliations:** 1 Universidad Santo Tomas, Bucaramanga, Colombia; 2 UNESCO Chair Sport for Development, Peace and Environment, Montreal, QC, Canada; 3 Université du Québec en Outaouais, Gatineau, QC, Canada; 4 Department of Psychology, Université Laval, Québec, QC, Canada; 5 Sciences de l'activité physique, Université du Québec à Montréal, Montréal, QC, Canada

**Keywords:** Colombia, disabled athletes, Latin America and Caribbean, paralympic, participation, sport for development, support

## Abstract

**Introduction:**

While Paralympic sport programs are increasingly recognized for their contribution to the well-being and social inclusion of people with disabilities, less attention has been given to the factors that facilitate or constrain athletes' participation within these programs. Understanding how individual motivation, support systems, and structural barriers influence engagement in sport programs is particularly relevant in under-researched contexts such as Latin America. This study aims to: a) describe the socio-demographic and sport participation profiles of athletes involved in a Paralympic sport training program in Bogotá, Colombia; b) examine the role of support systems—such as coaches, family members, and teammates—in shaping athletes' engagement in the program; and c) identify key motivations and barriers influencing sustained participation in Paralympic sport.

**Methods:**

A quantitative research design was used to collect data from athletes participating in the Paralympic sport training program implemented by the Instituto Distrital de Recreación y Deporte in Bogotá, Colombia. Data were gathered through a structured questionnaire examining athletes' demographic characteristics, sport participation history, sources of support, motivations for engagement in sport, and perceived barriers to participation. Descriptive and exploratory analyses were conducted to examine patterns of participation and program-related factors.

**Findings:**

Results indicate that athletes' engagement in the program is strongly supported by multiple social actors, particularly coaches, family members, and teammates. Coaching support emerged as a key enabling factor, with athletes reporting that encouragement and confidence expressed by coaches played an important role in sustaining their motivation. Athletes reported motivations related to improving physical well-being, enhancing appearance, and achieving sports performance goals such as winning medals. The predominance of men among participants also suggests that access to para sport may be shaped by intersecting forms of inequality, particularly those related to gender and disability.

**Discussion:**

The findings highlight the importance of supportive environments and institutional resources in facilitating sustained participation in Paralympic sport programs. These results underscore the need for policies and sport programs that address not only motivational and structural factors, but also intersecting forms of inequality, particularly those related to gender and disability, in order to expand equitable participation opportunities for people with disabilities in Colombia and similar contexts.

## 1 Introduction

### 1.1 Context of the study

An estimated 16% of the global population lives with some form of disability, representing more than 1.3 billion people worldwide (1). This portrait underscores the urgent need to implement concrete actions that ensure the full inclusion and participation of people with disabilities in all spheres of society. Despite notable progress over recent decades, substantial disparities persist between individuals with and without disabilities in terms of social participation, access to opportunities, and quality of life (1–4). As highlighted by the United Nations Convention on the Rights of Persons with Disabilities (CRPD), “persons with disabilities continue to face barriers that prevent them from participating in social life on an equal basis with others, and their fundamental rights continue to be violated in all regions of the world” (p. 2) (5). In response, the CRPD explicitly calls upon States Parties to adopt appropriate measures enabling people with disabilities to participate, on an equal basis with others, in recreational, leisure, and sporting activities (p. 22) (5).

Adapted sport initially emerged within rehabilitation contexts, where physical activity was used as a therapeutic tool, particularly for individuals with spinal cord injuries (6, 7). Over time, competitive sport became an extension of these rehabilitation programs, gradually moving beyond medical settings into more formalized and competitive sporting environments (8–10). In particular, Paralympic sport was initially developed under the leadership of health organizations and organizations of people with disabilities, rather than traditional sport institutions. As such, the Paralympic Movement was deeply rooted in a rights-based approach, advocating for inclusion, equity, and equal sporting opportunities for individuals with disabilities (9, 11).

Through close collaboration with the Olympic Movement, Paralympic sport progressively adopted similar training structures, competition formats, and performance standards. Today, the Paralympic Games stand as the second-largest multi-sport event in the world (12–14). Participation has increased dramatically, from only 16 athletes at the inaugural Stoke Mandeville Games in 1948, then to 400 in the first Paralympic Games in Rome in 1960, to more than 4,000 athletes competing at the Paris 2024 Paralympic Games [4,433 competitors (2,463 men and 1,970 women)], reflecting both the growth and institutionalization of Paralympic sport on a global scale^1^.

Paralympic athletes who reach elite levels of performance—whether by winning medals or qualifying for finals at world championships or the Paralympic Games—deserve recognition not only for their athletic talent, discipline, and rigorous training, comparable to their Olympic counterparts, but also for overcoming persistent cultural, social, and structural barriers (15, 16). These barriers often extend well beyond the sporting arena and can negatively influence both athletic development and broader social participation (17, 18). Indeed, qualifying for and competing in the Paralympic Games requires navigating a particularly challenging pathway, marked by limited resources, unequal access to training opportunities, and societal attitudes that continue to marginalize people with disabilities (15, 19, 20).

In this context, an intersectional perspective is also valuable for understanding how barriers to disability sport may differ among athletes (21). Intersectionality describes how multiple social identities and structures of inequality, including gender, disability, class, race, and sexuality, overlap and interact to produce different experiences of discrimination, exclusion, and privilege (22, 23). Research on the intersection of gender and disability shows that disabled women can experience overlapping forms of discrimination that are not reducible to either gender or disability alone (24). Research in sport suggests that sporting spaces may reproduce gendered, ableist, and heteronormative norms, which can affect who gains access to training, recognition, and competitive opportunities (21). Therefore, examining Paralympic pathways requires attention not only to structural and material barriers, but also to the ways in which disability intersects with gender and other social inequalities to shape participation and advancement in sport (21, 24).

### 1.2 Relevance of the study in LAC

Globally, participation in physical activity and sport remains unevenly distributed among persons with disabilities, reflecting broader structural inequalities related to health, education, and social inclusion. In Latin America and the Caribbean (LAC), the prevalence of disability is comparable to global estimates, with approximately 14.7% of the population—around 85 million people—living with some form of disability (25). Despite this substantial proportion, access to organized physical activity and sport remains limited for many individuals with disabilities across the region, particularly in low- and middle-income contexts (25).

Socioeconomic vulnerability further compounds these inequalities. Evidence from the World Bank indicates that households including persons with disabilities are disproportionately affected by poverty, with nearly seven out of ten such households considered at risk of falling into poverty (25). These socioeconomic constraints directly influence access to sport and physical activity by limiting opportunities related to transportation, equipment, specialized coaching, and access to facilities designed or adapted for this population that provide safe sport environments. As a result, sport participation for people with disabilities in LAC often depends on targeted public policies and institutional support rather than on individual or family resources alone.

Recent outcomes from the Paris 2024 Paralympic Games illustrate notable disparities in the level of elite sport development within the LAC region. Brazil emerged as a global leader, finishing fifth in the overall medal rankings, followed by Colombia (19th), Cuba (24th), and Mexico (30th). Other countries, including Argentina (34th), Ecuador (47th), Costa Rica (49th), Chile (60th), and Peru (65th), ranked further down the standings^2^. These results highlight considerable variation in the capacity of national systems to support high-performance Paralympic athletes.

While competitive results should not be interpreted as the only direct or comprehensive indicator of progress in physical activity and sport participation among persons with disabilities, they nonetheless provide insight into the broader level of institutional investment, infrastructure, and policy development within a given country. In this sense, Paralympic performance can be understood as one visible outcome of the social, economic, and organizational conditions that shape opportunities for persons with disabilities to engage in sport. Therefore, examining national and local sport programs remains essential to understanding how sport can contribute to inclusion, well-being, and quality of life in diverse social contexts, such as Colombia. Thus, intersectionality as mentioned previously is also another layer to consider regarding the context of LAC (21).

### 1.3 National and local context of the IDRD program

Over the past several decades, Colombia has progressively developed a policy framework aimed at promoting the inclusion of persons with disabilities through physical activity, sport, and recreation. The Ministry of Sport—formerly Coldeportes—has played a central role in integrating Paralympic sport into the national sport system, not only as a high-performance pathway but also as a mechanism for social inclusion^3^.

A key component of Colombia's disability sport policy is the principle of equity between the Olympic and Paralympic sectors according to which incentives for athletes with disabilities must be equivalent to those provided to Olympic athletes at local and national levels. This includes access to supported athletic programs, incentives for medallists at national and international competitions, and long-term support for athletes identified as future leaders in sport for people with disabilities. This policy orientation reflects a rights-based approach that seeks to reduce structural inequalities within the national sport system.

Within this institutional framework, Paralympic sport in Colombia has experienced significant development over the past two decades. Initially driven by organizations representing people with disabilities, leadership has progressively expanded to include a broader range of sports entities associated with the Paralympic Movement. These include integrated Olympic federations, Paralympic sport federations, the Colombian Paralympic Committee, and specialized sport organizations for people with disabilities. A major milestone in this development was the organization of the first Para National Games in 2004, held alongside the 17th National Games. Since then, the Para National Games have been conducted every four years in parallel with the National Games, following the international Paralympic model. This event, which brings together athletes from Colombia's political and administrative regions, has played a crucial role in strengthening the national Paralympic sport structure.

At the local level, there is an institution responsible for recreation and sport - *District Institute of Recreation and Sport* (Instituto Distrital de Recreación y Deporte; IDRD) - which acts as the political, legal, methodological, and strategic authority guiding sport development in the city. Within this framework, Paralympic sport is identified as a core component of the institution's mission. The local sport organization has established a comprehensive system of support and incentives designed to ensure adequate conditions for athlete development across multiple stages of performance, from entry-level participation to high-performance sport.

Support is structured according to performance level and participation in national and international events and includes access to sport facilities, qualified coaches, multidisciplinary support teams, competition-related assistance, and financial incentives. In addition, there is a psychosocial support component aimed at addressing the specific needs of athletes facing social vulnerability, particularly those living in contexts of economic deprivation or social risk. Ultimately, this program combines athletic performance goals with broader goals of social inclusion and well-being.

### 1.4 Research objectives

This study pursues three complementary objectives:

1. To describe the socio-demographic, disability-related, and sport participation profiles of athletes involved in the IDRD Paralympic sport training program;
2. To examine the role of support systems in shaping athletes’ engagement in the sport program;
3. To identify barriers and enabling factors affecting sustained participation in Paralympic sport.

## 2 Methods

### 2.1 Design and procedure

A quantitative method was designed for this project. Data was collected through an online survey with *Lime Survey*, which took about 20 min to complete. Participants provided consent before completing the survey and completed it mostly on their cellphones between October 2024 and June 2025. Completion was realized in collaboration with coaches of the different parasport teams in order to select an adequate spot to explain the project to the athletes, let them participate if they so wished, without interfering with training sessions.

### 2.2 Participants

The 105 participants, Colombian athletes with disabilities, were aged between 14 and 59 years old. They all trained and competed in the Paralympic sector of the local sport organization (IDRD) in Bogotá, in different para-athletic disciplines. Subjects in the study participated on a voluntary basis, and they were recruited by those responsible for the organization (e.g., administrators, coaches, peers). Before the completion session, participants received an invitation letter by email or in person, describing the project. To be included in the study, participants were required to be registered in a specific sport training group run by the local sport organization (IDRD focused on performance and competition and led by a professional coach who was, in turn, supervised by experts in sport training methodology). Subjects in the study participated on a voluntary basis, and they were invited to participate through administrators, coaches, or fellow athletes. Depending on their personal abilities and disabilities, athletes completed alone with their own cellphone or with help (e.g., oral completion with assistant) the survey.

### 2.3 Measures

The self-administered questionnaire in Spanish was specifically developed for the purpose of this study. Questions were developed by the research team (SR, MG, TG) based on initial reflections by the first author with his IDRD colleagues. The tool was pilot tested^4^ with 10 athletes from the program before being released online. The instrument included open-ended questions, dichotomous yes-no questions and items assessed using a 5-point Likert scale ranging from 1 (“strongly disagree”) to 5 (“strongly agree”). It assessed socio-demographic and disability characteristics, attributes of the sport program and perceived benefits of sport. An entire version (in Spanish) of the questionnaire is available (Supplementary Appendix A).

### 2.4 Data analysis

Data collected with the online questionnaire was preprocessed and then analyzed with *IBM SPSS Statistics* (version 31; IBM Corp., 2025^5^)*.* Descriptive statistics (mean, standard deviation, frequency, and percentage) were used to address the study aims. Due to occasional missing responses, sample sizes varied slightly across analyses and figures. Percentages were calculated based on valid responses for each item.

Due to occasional missing responses, the number of valid responses (N) varied slightly across questionnaire items. Therefore, percentages were calculated based on valid responses for each item, and the corresponding valid sample sizes are reported in Tables 1, 2 to improve transparency and consistency across analyses.

**Table 1:** Socio-demographic characteristics of participants and description of handicap (*N* = 105).

Characteristics	*N*	*%*
Age		
Youth (13–17 yo)	12	11.4
Young adult (18–25 yo)	39	37.1
Adult (25 yo +)	54	51.4
Gender		
Female	26	25.0
Male	78	75.0
Handicap type		
Physical	68	66.0
Visual	24	23.3
Cognitive	6	5.8
Auditory	5	4.9
Start of handicap		
Birth	54	52.9
Childhood and youth	27	26.5
Adulthood	21	20.6

Sample size varied due to missing values.

**Table 2:** Participants’ athletic profile (*N* = 105).

Categories	*N*	*%*
Sport		
Para Swimming	21	20.2
Goalball	13	12.5
Para Athletics	12	11.5
Wheelchair Rugby	9	8.7
Wheelchair Basketball	3	2.9
Para Table Tennis	8	7.7
Weightlifting	11	10.6
Football 5-A-Side	9	8.7
Bowling	1	1.0
Football 7-A-Side	11	10.6
Para Cycling	6	5.8
Start of current sport participation, *years*		
<1	7	6.7
1–2	12	11.4
3–4	17	16.2
5–6	10	9.5
7–8	15	14.9
>8	44	41.9
Weekly training sessions frequency		
1–2	5	4.8
3–4	27	25.7
5–6	36	34.3
7–8	12	11.4
9–10	14	13.3
>10	11	10.5
Competition level		
None	7	6.7
Regional tournaments	4	3.8
National championships	42	40.4
Authorized international championships National Federation	9	8.7
Parapan American Games	16	15.4
World championships	12	11.5
Paralympic Games	14	13.5
Past participation in sports		
Yes	54	51.4
No	51	48.6
Training initiation age		
<11	17	16.2
11–12	13	12.4
13–14	6	5.7
15–16	19	18.1
17–18	10	9.5
>18	40	38.1

Sample size varied due to missing values.

Also, because some subgroup analyses were based on relatively small sample sizes, these descriptive findings should be interpreted cautiously and viewed as exploratory tendencies rather than robust group differences.

For Supplementary Figure S1 (Supplementary Appendix B), average scores were calculated by computing the arithmetic mean of participants' responses for each dimension assessed in the questionnaire. Measures of variability, including standard deviations, were added to improve the interpretation and transparency of the descriptive results. These scores should be interpreted as exploratory descriptive indicators rather than standardized or validated composite scales. Because some participants did not answer all questionnaire items, the number of valid responses differed slightly between dimensions and analyses.

### 2.5 Ethics

The study design and procedures were approved by the Université du Québec à Montréal Ethics Committee (Approval number: 2024-6258).

## 3 Results

### 3.1 Socio-demographic, disability-related, and sport participation profiles of athletes involved in the IDRD paralympic sport training program

The first objective documents athletes’ characteristics, including age, gender, type and onset of disability, sport practised, level of competition, training frequency, and previous sport experience.

### 3.2 Socio-demographic and disability-related profiles of athletes

The socio-demographic and disability-related characteristics of the sample are presented in Table 1. Participants had a mean age of 28 years (*SD* = 10.35), and the majority were men (75%). Approximately half of the participants reported a congenital or genetic disability present since birth, whereas the remaining individuals acquired their disability later in life. Most participants (64.8%) had a physical disability, while the others presented visual (23%), cognitive (5.7%) or auditory (4.8%) impairments.

### 3.3 Sport participation profiles of athletes

Table 2 summarizes the participants' athletic characteristics. The majority were engaged in para swimming, goalball and para-athletics. Overall, 41.9% of participants had been involved in their current sport for more than eight years, while 69.5% reported training five or more times per week. Competition levels ranged from regional events to world championships and Paralympic Games. Approximately half of the participants had practised a sport before joining the current program, and 38.1% began training after the age of 18.

Regarding gender, a substantial proportion of women reported 7-8 years of sport participation (42.3%), and nearly half of men reported more than 8 years of experience (46.8%). Male athletes were evenly distributed across the range of para-sports, whereas female athletes were primarily concentrated in swimming (8/26; 30.8%) and goalball (7/26; 26.9%). Most athletes competed at the national championship level, including 34.6% of women (9/26) and 40.5% of men (32/78). A greater proportion of women competed at higher-level events, including the Parapan American Games (26.9% of women vs. 11.4% of men) and the Paralympic Games (19.2% vs. 11.4%).

### 3.4 Perceived impacts of disability by the athletes

**Figure 1:**
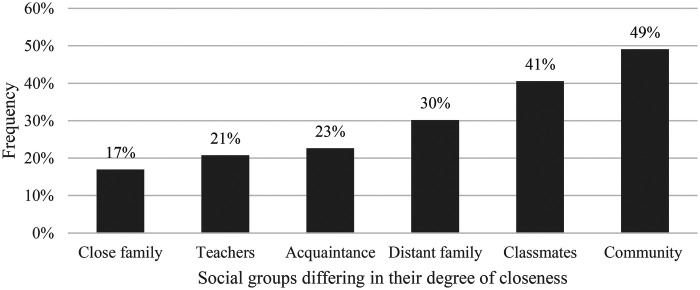
Does having a disability lead to people being socially excluded or being a victim of prejudice from social groups? perceptions of para-athletes (*N* = 105).

Participants were asked to evaluate the perceived impact of their disability. Figure 1 presents the reported experiences of prejudice and exclusion across social relationships differing in intimacy level. Overall, participants perceived greater judgment from more distant social groups such as the community, classmates, and distant relatives, and less from closer relationships with immediate family, teachers and acquaintances.

**Figure 2:**
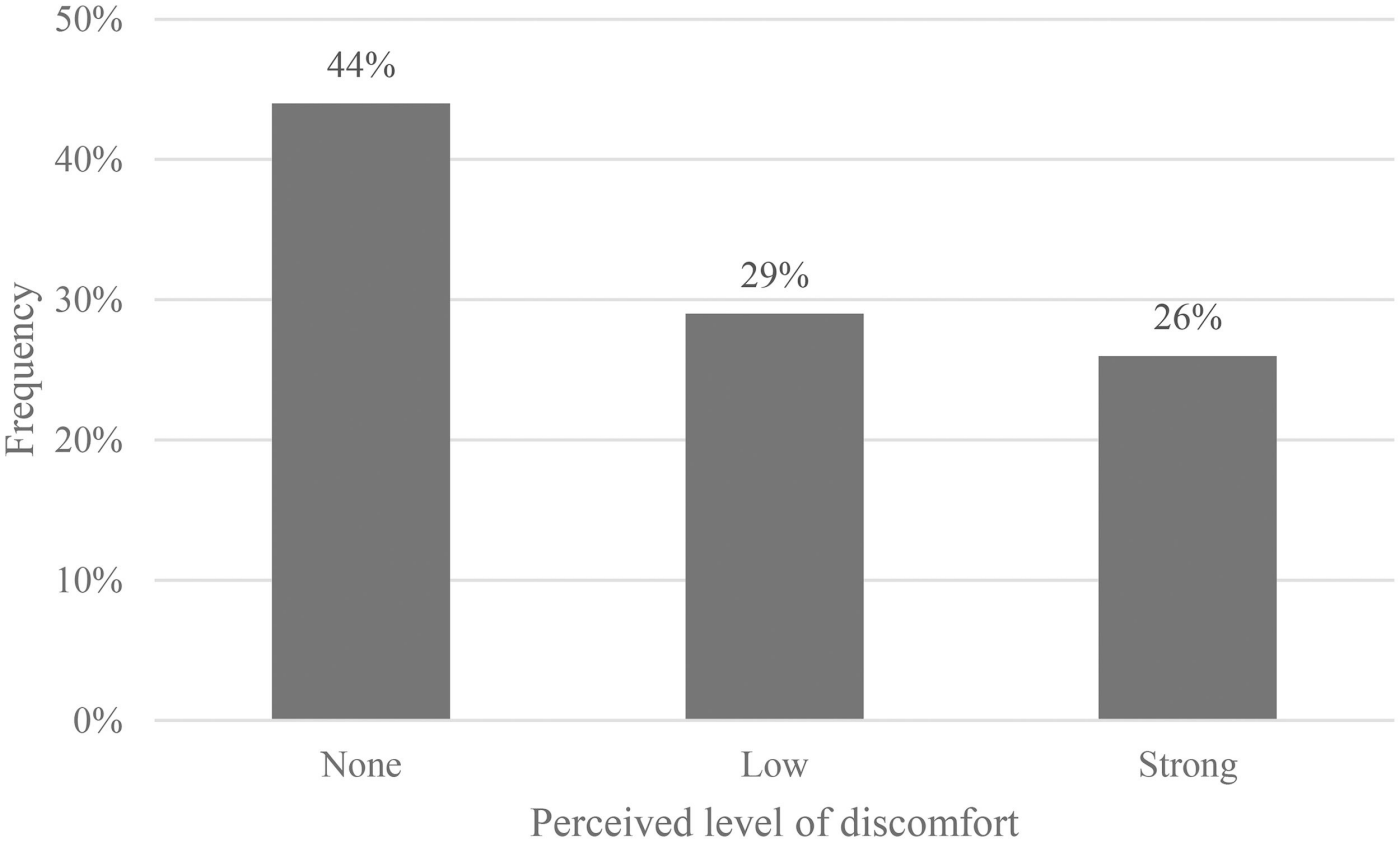
Para-athletes’ subjective discomfort when their disability is noticed by others.

Generally, 52.8% of participants indicated that their disability negatively impacted their self-esteem. Although this perceived effect on self-esteem is significant, disability does not appear to greatly limit participation in various activities. Regarding the impact of disability on activity participation, 60.4% of respondents strongly agreed that it did not influence their sporting performance, while 53.8% strongly agreed it did not limit other aspects of their daily lives. Participants’ reactions varied regarding the level of discomfort felt when others noticed their disability: 44% reported no discomfort, 29% experienced low levels of discomfort, and 26% reported strong discomfort. These results are illustrated in Figure 2.

Depending on types of handicaps, some tendencies could be noticed. First, para-athletes with physical impairments appeared to engage in a broader range of sports, including swimming, athletics, rugby, basketball, table tennis, weightlifting, football and cycling. In contrast, para-athletes with visual impairments participated in a more limited set of sports, primarily swimming, goalball, athletics, football and bowling. Those with cognitive or auditory impairments seemed to have the most restricted participation, with swimming, athletics and cycling.

Second, for para-athletes with cognitive or auditory disabilities, the majority reported prior participation in other sports before enrolling in the current sport program, a proportion higher among athletes with cognitive (66.7%) and auditory (60.0%) disabilities compared to those with physical (48.5%) or visual (54.2%) impairments. Half of the participants with a cognitive handicap (50%) reported first engaging in sport before the age of 11 years, compared to approximately 10%–20% of participants with other types of handicaps.

Third, para-athletes with cognitive impairments reported the highest levels of perceived support across several sources, including family members (M = 5.00, SD = 0), coaches (M = 5.00, SD = 0), school (M = 3.17, SD = 1.83), and acquaintances (M = 3.17, SD = 1.83), compared with participants with other types of impairments. In contrast, para-athletes with physical impairments generally reported lower levels of support than other participants, particularly from school (M = 2.55, SD = 1.5), coaches (M = 4.41, SD = 0.96), acquaintances (M = 2.42, SD = 1.44), and IDRD officials (M = 3.62, SD = 1.27). Para-athletes with visual impairments reported the highest levels of support from teammates (M = 4.61, SD = 0.66) and IDRD officials (M = 4.09, SD = 1.24). In addition, an important proportion of participants with visual impairments (78.3%) reported that their disability affected their self-esteem, compared with approximately 40%–50% among participants with other impairments.

Finally, participants with physical and auditory handicaps reported perceived social exclusion or prejudice from the community, respectively 70.9% and 75%. In contrast, approximately 40% of participants with visual or cognitive disabilities indicated experiencing such challenges. All participants with cognitive (6/6) or auditory (5/5) impairments reported improved self-esteem since participating in a sport, compared with 21/23 (91.3%) of participants with visual impairments and 49/57 (70.9%) of those with physical disabilities.

### 3.5 Support systems in shaping athletes' engagement in the IDRD paralympic sport training program

**Figure 3:**
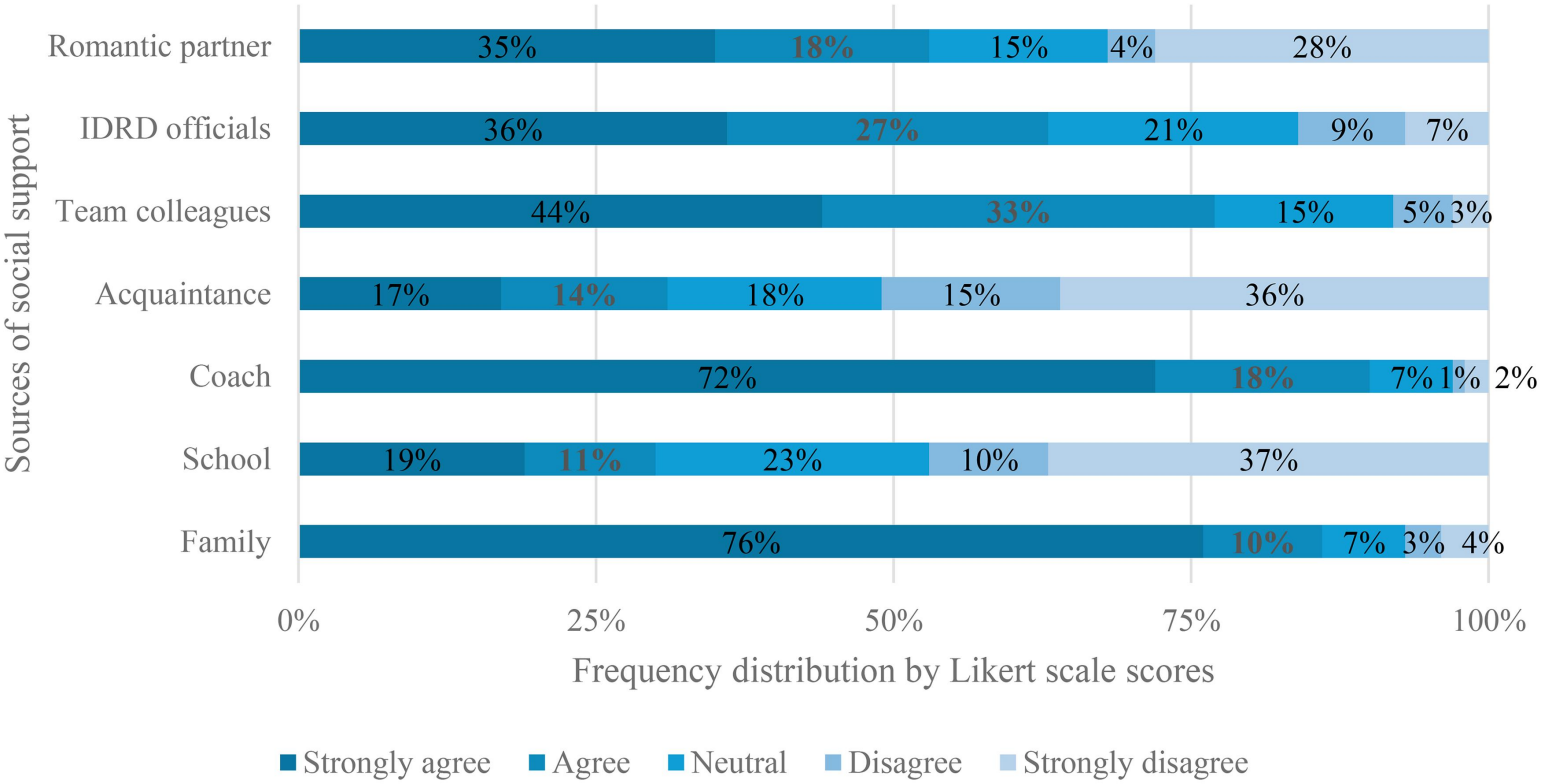
Para-athletes’ perceptions of support from multiple social groups related to sport involvement.

This second objective analyzes the influence of key sources of support, including coaches, family members, and teammates, as well as athletes' motivations for participating in the program. Perceived support for sport participation varied depending on the social group. In Figure 3, the frequency distribution of Likert scale scores is presented for each source of social support with the most support provided by family members, coaches and team members. Athletes reported less support from school and acquaintances.

With regard to parental support, specifically, 83% of participants reported the highest possible score for perceived support from their parents. Additionally, coaches represented a major source of support for para-athletes, as they were individuals with whom athletes spent most of their daily time. Regarding age groups, youth athletes aged 13–17 reported lower levels of perceived support from their romantic partner (M = 2.09, SD = 1.70) on a 5-point Likert scale (1 = strongly disagree; 5 = strongly agree), whereas adults aged 25 years and older reported higher levels of support (M = 3.64, SD = 1.56). For school-related support, youth (13–17 years) and young adults (18–25 years) reported mean scores of 2.91 (SD = 1.51) and 3.06 (SD = 1.39), respectively, compared to a lower mean score of 2.32 (SD = 1.58) among adults aged 25 years and older.

**Figure 4:**
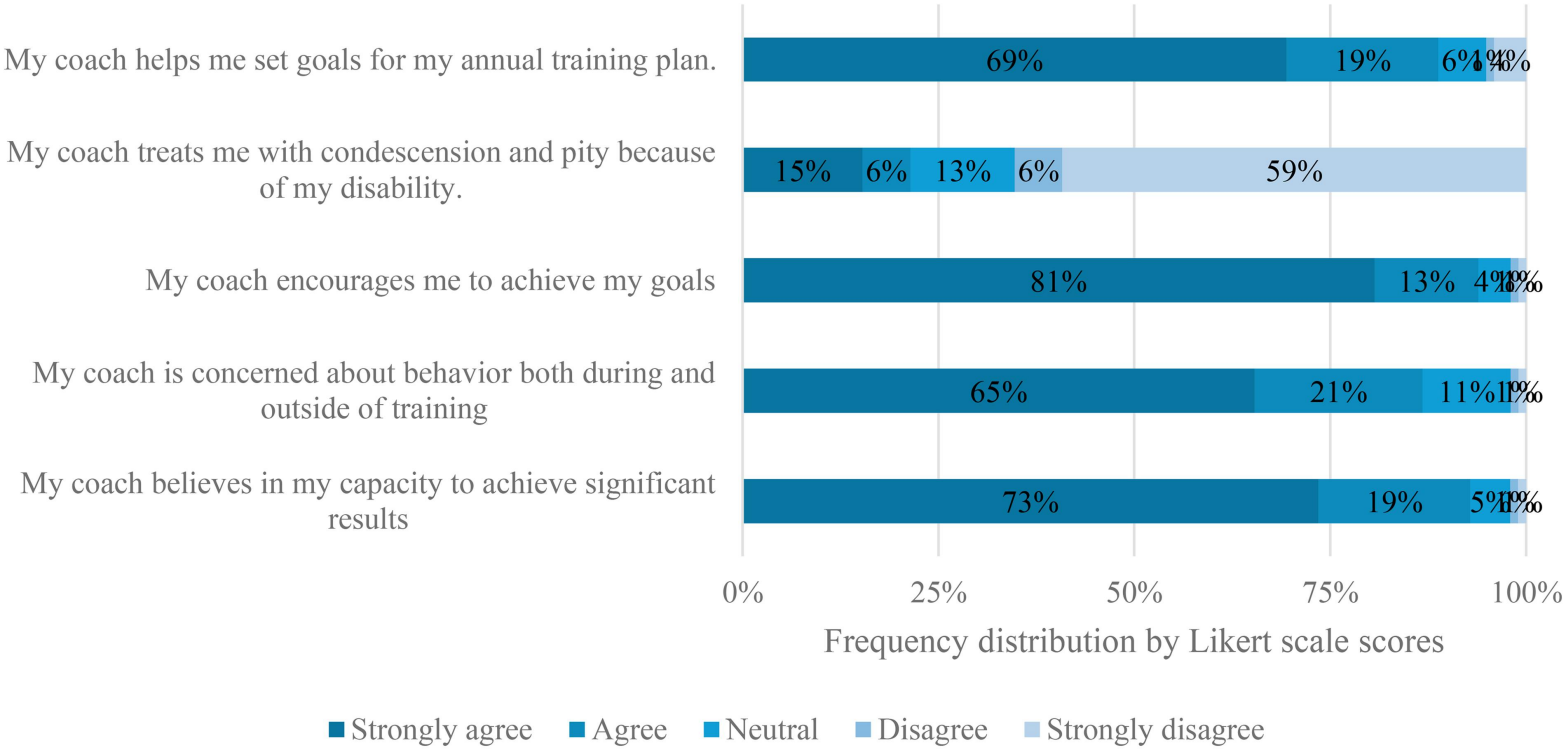
Perceived coach supportive behaviors and attitudes by para-athletes.

As illustrated in Figure 4, the frequency distribution of Likert scale responses highlights supportive attitudes and behaviors from coaches. Notably, coaches were perceived as a key motivational influence in achieving personal sport-related goals as well as conveying confidence and trust in their athletes. However, it is worth mentioning that a non-negligible proportion of athletes strongly agreed or agreed with the statement indicating their coach treated them with pity or condescension^6^.

### 3.6 Barriers and enabling factors affecting sustained participation in the IDRD paralympic sports training program

**Figure 5:**
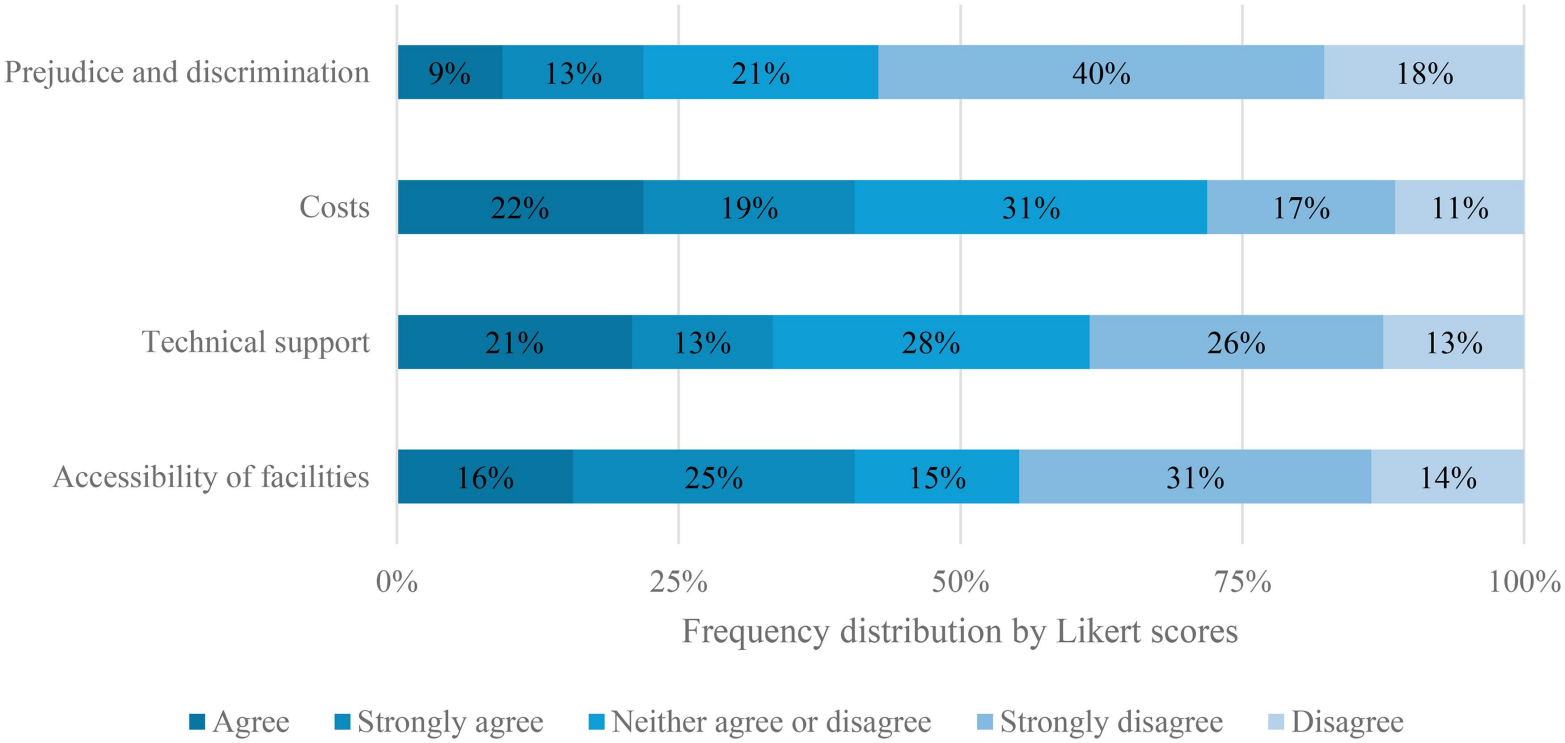
Factors limiting participation in sport training programs.

This third and last objective focuses on structural and contextual factors such as financial constraints, access to resources, and program-related support mechanisms. The participants’ responses suggest that financial constraints and insufficient technical support represented the primary barriers to engagement in the sport program. Figure 5 illustrates the frequency distribution of responses across different barrier categories.

## 4 Discussion

### 4.1 Major findings

The present study aimed to better understand the determinants of participation and support mechanisms within a Paralympic sport training program implemented by the District Institute of Recreation and Sport (IDRD) in Bogotá, Colombia. Three main findings emerge from the results.

First, the socio-demographic and sport participation profiles of the athletes reveal a relatively experienced and committed group of participants. Most athletes reported several years of involvement in their sport and high training frequencies, with a large proportion training five or more times per week. Additionally, athletes were engaged across multiple competitive levels, including national championships, Parapan American Games, world championships, and even the International Paralympic Games. These findings suggest that the implementation of appropriate policies from the program administration, which provides a structured environment capable of supporting athletes across different stages of development, from entry-level participation to high-performance sport, coincided with the facilitators previously identified in the literature (16, 19).

Second, the results highlight the complex relationship between disability and social participation. While many athletes reported experiences of exclusion or prejudice in certain social environments (17, 18)—particularly within the broader community—most participants indicated that their disability did not strongly limit their sport participation or other areas of daily life. These findings suggest that sport participation may play a protective or empowering role in the lives of people with disabilities by providing opportunities for social recognition, identity construction, and personal development (3, 17). However, given the descriptive and cross-sectional nature of the present study, these findings should be interpreted cautiously. The data do not allow causal conclusions regarding the direct effects of sport participation on empowerment, inclusion, or psychosocial development. Rather, the results highlight perceived associations reported by participants within the context of the IDRD program. In addition, the predominance of men (75%) among participants also points to the complexity of inclusion in para sport. From an intersectional perspective, this gender imbalance suggests that access to sport may be shaped not only by disability, but also by gendered forms of discrimination and broader structural inequalities. From an intersectional perspective, this gender imbalance may reflect broader social and structural dynamics shaping access to para sport. Although the present study was not specifically designed to examine intersectional mechanisms, the descriptive findings are consistent with literature suggesting that disability and gender can interact to influence sport participation opportunities (21, 24). Therefore, the lower representation of women in the program may reflect not only individual preferences or sport trajectories, but also the cumulative effects of social, cultural, and institutional barriers that limit women's participation and advancement in disability sport. This finding is also consistent with critical disability studies in sport, which argue that gender and disability norms intersect in ways that may reproduce ableist, heteronormative, and gendered patterns of exclusion (21).

Third, results indicate that multiple social actors contribute to athletes' engagement in the program. Coaches, family members, and teammates appear to play a central role in sustaining motivation and participation. In particular, athletes emphasized the importance of coaching support, encouragement, and trust in their abilities (26–28). These relational dimensions suggest that participation in Paralympic sport programs is shaped not only by individual motivation but also by the broader social environment surrounding athletes (29).

### 4.2 Elements of discussion

The findings of this study contribute to the growing literature highlighting the importance of social and environmental determinants in shaping participation in sport among people with disabilities (26, 27, 29). Previous research has shown that barriers to participation are often linked to structural factors such as accessibility, financial constraints, and social attitudes toward disability (16, 27). In the present study, athletes reported experiences of social exclusion in several contexts, particularly within the broader community. These findings align with earlier work, demonstrating that individuals with disabilities continue to face stigma, prejudice, and social marginalization despite increasing policy commitments to inclusion (2, 17, 19), with evidence suggesting that these forms of marginalization may be further intensified when gender is taken into account.

At the same time, the results suggest that participation in structured sport programs may mitigate some of these negative social experiences (16, 19, 28), and it is an important result for the LAC context where discrimination could be exacerbated. Athletes reported that their disability did not substantially limit their sport participation or other life domains, and many described positive perceptions of their abilities and future life prospects. This observation supports previous studies emphasizing the empowering potential of sport participation for people with disabilities (11). Through sport, individuals may develop confidence, social networks, and a sense of belonging that counterbalance experiences of exclusion encountered in other areas of social life, as already reported previously (14, 19, 26), as well as in this study. While the present data do not directly assess these theoretical processes, crip theory nevertheless offers a useful interpretative lens for situating the findings within broader discussions concerning disability, visibility, and inclusion in sport contexts. This perspective highlights how sport can both reflect and reinforce dominant norms, while also offering possibilities to challenge them by increasing the visibility, recognition, and inclusion of diverse disabled bodies and identities. Although this literature is largely conceptual, it is useful for interpreting gender disparities in participation and for understanding how multiple systems of inequality may operate simultaneously in para-sport contexts (21).

Another important contribution of this study concerns the role of support systems in sustaining athletes' engagement in sport. Consistent with previous research on coach–athlete relationships in disability sport (30), the results indicate that coaches play a key role not only in athletic development but also in motivation, confidence building, and long-term commitment to sport (27, 28). Similarly, family support emerged as an important enabling factor, reflecting the broader social context in which athletes' sport participation takes place.

This study recalled that financial constraints also appear to represent a significant barrier to participation (31). Economic barriers are frequently identified in studies examining sport participation among people with disabilities, particularly in low- and middle-income contexts where access to specialized equipment, transportation, and training resources may be limited. In the LAC region, where households including people with disabilities are disproportionately exposed to poverty (25), these structural barriers may significantly shape opportunities for sustained participation in sport.

Taken together, these findings reinforce the importance of adopting a multidimensional perspective when examining participation in Paralympic sport programs. In this sense, the IDRD sport program proposed an interesting model to inspire other sport organizations in Global South or from LAC contexts. Participation is influenced not only by individual motivation or physical ability but also by a complex interplay of social support, institutional structures, and broader socioeconomic conditions.

### 4.3 Limitations and strengths

This study presents several limitations that should be acknowledged. First, the data rely on self-reported responses collected through a questionnaire, which may introduce social desirability bias. It is possible that participants may have tended to report more positive experiences or perceptions of the program. Second, the voluntary nature of participation may have produced a selection bias, as athletes who chose to complete the survey may have been those most engaged in the program. Third, the relatively long questionnaire may have influenced response patterns or completion rates that could explain variations in total number for each answer (see Tables 1, 2).

Despite these limitations, the study also presents several strengths. One of its main contributions lies in capturing the subjective experiences of Paralympic athletes participating in a structured sport program in a LAC context, an area that remains underrepresented in the literature (32–34). The study also provides insight into community-based and developmental sport programs rather than focusing exclusively on elite sport performance pathways. Finally, the research offers a multidimensional perspective by examining not only sport participation but also the broader social and relational factors that shape athletes' experiences, which we think is crucial, considering the role of sport for development in disabled athletes (35).

In addition, some analyses were conducted with varying sample sizes due to missing responses across questionnaire items. As a result, percentages and frequencies occasionally differ slightly between analyses and tables. To improve transparency, the valid number of responses for each variable was systematically reported where applicable.

Given the exploratory nature of the study, the analyses primarily relied on descriptive statistics. Therefore, the findings should be interpreted as indicative tendencies within this specific sample rather than as generalizable conclusions applicable to all Paralympic athletes or sport programs in Colombia or the broader LAC region.

## 5 Conclusion

This study examined the determinants of participation and support mechanisms within a Paralympic sport training program implemented by the local sport organization (IDRD) in Bogotá, Colombia. The results highlight the importance of structured sport environments that combine athletic development with strong social support networks. Coaches, family members, and teammates play a central role in sustaining athletes' engagement, while financial constraints remain an important barrier to participation. The predominance of men among participants also suggests that gender may shape access to and progression within Paralympic sport, highlighting the need for an intersectional approach that considers how disability, gender and other social axes of inequality interact to produce unequal participation opportunities.

Beyond sport performance, the findings suggest that athletes perceived participation in Paralympic sport programs as being associated with broader processes of empowerment and social participation. Even when individuals experience social exclusion in certain contexts, sport participation may provide opportunities for identity construction, recognition, and personal development.

In the context of LAC, where structural inequalities and socioeconomic vulnerabilities continue to shape access to sport, programs such as the one referenced in this study play an important role in expanding participation opportunities for people with disabilities. Strengthening these programs through supportive policies, accessible infrastructures, and sustained institutional investment may, therefore, represent an important pathway toward greater inclusion and well-being.

Future research could further explore how different program characteristics—such as coaching approaches, training environments, or institutional support mechanisms—shape long-term participation and developmental outcomes for Paralympic athletes. Longitudinal studies would also be valuable to help better understand how sport participation influences life trajectories for people with disabilities over time.

Although the present findings provide valuable insight into athletes' experiences within the IDRD program, future research using longitudinal and comparative designs would be necessary to better understand the long-term impacts of participation and the specific mechanisms through which sport programs may influence inclusion, well-being, and life trajectories.

## Data Availability

The raw data supporting the conclusions of this article will be made available by the authors, without undue reservation.

## Footnotes

^1^ https://www.paralympic.org/paris-2024/results

^2^ https://www.paralympic.org/paris-2024/results

^3^Political Constitution of Colombia, 1991; Law 1618, 2013. Available here: https://www.funcionpublica.gov.co/eva/gestornormativo/norma.php?i=52081

^4^The questionnaire did not undergo formal psychometric validation, because questions were created to serve the specific intentions of the researchers’ teams.

^5^IBM Corp. (2025). *IBM SPSS Statistics for Windows* (Version 31.0) [Software]. https://www.ibm.com/products/spss-statistics

^6^The statement evaluating condescending or pitying treatment by coaches was negatively worded, in contrast to the preceding and subsequent items. This reversed wording may have introduced confusion among participants and affected their response for that precise item.
