# Supplementary material for: Determinants of participation and support mechanisms in a paralympic sport training program: evidence from the IDRD program in Bogotá
Source: Front Sports Act Living. 2026 Jul 3;8:1882854. doi: 10.3389/fspor.2026.1882854 (PMC13377818; doi:10.3389/fspor.2026.1882854)
Supplement: Supplementary file 1 [file Supplementaryfile1.docx]

**APPENDIX A – INCUESTA (Survey in Spanish)**

**Beneficios de un programa de entrenamiento deportivo paralímpico en la participación de los atletas (IDRD - Colombia)**

**Introducción**

Descripción proyecto + objetivo

Formulario de consentimiento

1. **Perfil del participante**
   1. Edad cronológica (Hasta que cumplea)

14

15

16

17

18

19

20

21

22

23

…

1.2 Tipo de discapacidad Física

- Visual
- Cognitiva
- Limitación auditiva
  1. Desde cuándo tiene esta discapacidad

________________________________

- 1. Género
- Masculino
- Femenino
- Otro
- NS/NR
  1. Hace cuanto practica el deporte actual
- Menos de 1 año
- 1-2 años
- 3-4 años
- 5-6 años
- 7-8 años
- + de 8 años
  1. Deportes que practicas

-para natación

-gol bol

-Atletismo

-rugby cila de ruedas

-balón cesto

-tenis de mesa

-Pesas

-Futbol 5

-otro: ________________

1.7 Cuantas veces por semana entrena o practica el deporte actual

- 1-2
- 3-4
- 5-6
- 7-8
- 9-10
- + de 10

1.7 ¿Ha practicado otros deportes en el pasado? Si es así, ¿cuáles?

1.8 Edad de inicio de entrenamiento

- Antes de los 11 años
- 11-12 años
- 13-14 años
- 15-16 años
- 17-18 años
- + de 18 años

1.9 Nivel competitivo (Marcar solamente el máximo nivel en que ha competido)

- No he competido aún
- Torneos regionales
- Campeonatos nacionales
- Campeonatos internacionales autorizados por la federación local respectiva
- Juegos Parapanamericanos
- Campeonatos mundiales
- Juegos Paralímpicos

1.10 ¿Cuánto tiempo le dedicas en la semana a las siguientes actividades? Marca con una X la opción escogida.

|  | 0-2 Horas | 2-4 Horas | 4-6 Horas | 6-8 Horas | 8-10 Horas |
| --- | --- | --- | --- | --- | --- |
| 1. Entrenando |  |  |  |  |  |
| 1. Paseando en la calle |  |  |  |  |  |
| 1. Jugando en el computador |  |  |  |  |  |
| 1. Leyendo |  |  |  |  |  |
| 1. Viendo televisión |  |  |  |  |  |
| 1. Estudiando en una institución |  |  |  |  |  |
| 1. Estudiando en casa |  |  |  |  |  |
| 1. Haciendo nada |  |  |  |  |  |

1. **Percepción del deporte para el participante**

2.1 ¿Qué tan importante es para ti la participación en el grupo de deporte?

- Muy importante
- Es importante
- Poco importante
- Nada importante
- No sabe no responde

2.2 ¿Qué te motiva a participar en deporte?

Por favor califique de 1 a 5 todas las opciones de respuesta en donde 1 es el valor mínimo de influencia y 5 el máximo valor.

| 1. Mejorar la salud | 1 | 2 | 3 | 4 | 5 |
| --- | --- | --- | --- | --- | --- |
| 1. Sentir la emoción de la competencia sin importar los resultados | 1 | 2 | 3 | 4 | 5 |
| 1. Compartir con los amigos | 1 | 2 | 3 | 4 | 5 |
| 1. Ganar medallas | 1 | 2 | 3 | 4 | 5 |
| 1. Obtener los incentivos económicos que otorga el IDRD o el gobierno nacional | 1 | 2 | 3 | 4 | 5 |
| 1. En tu casa quieren que tú participes | 1 | 2 | 3 | 4 | 5 |
| 1. Viajar y conocer ciudades y gente | 1 | 2 | 3 | 4 | 5 |
| 1. Te ves o te sientes mejor físicamente | 1 | 2 | 3 | 4 | 5 |
| 1. Te sientes mejor emocionalmente | 1 | 2 | 3 | 4 | 5 |
| 1. Te sientes incluido socialmente | 1 | 2 | 3 | 4 | 5 |

1. **Relaciones interpersonales**

Por favor marca con una x la opción que más te parezca conveniente.

- 1. Debido a tu discapacidad, alguna vez experimentaste trato excluyente, condescendiente o lastimero por parte de:

|  | SI | NO | NR |  |
| --- | --- | --- | --- | --- |
| 1. Familia cercana (Padres, hermanos) |  |  |  |  |
| 1. Familia extensiva (Tíos, tías, primos, primas, abuelos) |  |  |  |  |
| 1. Compañeros del colegio |  |  |  |  |
| 1. Profesores del colegio |  |  |  |  |
| 1. Amigos y conocidos del barrio |  |  |  |  |
| 1. Comunidad en general |  |  |  |  |

3.2 ¿Consideras que tu discapacidad ha sido una limitante para relacionarte con:

|  | SI | NO | NR |
| --- | --- | --- | --- |
| 1. Familia cercana (Padres, hermanos) |  |  |  |
| 1. Familia extensiva (Tíos, tías, primos, primas, abuelos) |  |  |  |
| 1. Compañeros del colegio |  |  |  |
| 1. Profesores del colegio |  |  |  |
| 1. Amigos y conocidos del barrio |  |  |  |
| 1. Comunidad en general |  |  |  |

3.3 ¿Alguna vez te sentiste afectado (a) en tu autoestima por tener una discapacidad?

- Si
- No
- NR

3.4 Desde que practicas deporte consideras que ha mejorado el trato de:

|  | SI | NO | NR |
| --- | --- | --- | --- |
| 1. Familia cercana (Padres, hermanos) |  |  |  |
| 1. Familia extensiva (Tíos, tías, primos, primas, abuelos) |  |  |  |
| 1. Compañeros del colegio |  |  |  |
| 1. Profesores del colegio |  |  |  |
| 1. Amigos y conocidos del barrio |  |  |  |
| 1. Comunidad en general |  |  |  |

1. **Percepción de la amistad y conexión con la comunidad**

4.1 ¿Consideras que en tu vida futura seguirás practicando deporte?

- Definitivamente lo haré
- Probablemente lo haré
- Quizás si Quizás no
- Probablemente no lo haré
- Definitivamente no lo haré

4.2 ¿A tus padres les gusta que practiques deporte?

- Completamente de acuerdo
- Parcialmente de acuerdo
- Les es indiferente
- Parcialmente en desacuerdo
- Totalmente en desacuerdo

4.3 Desde que practicas deporte crees que eres más ordenado (a) y disciplinado (a)

- Completamente de acuerdo
- Parcialmente de acuerdo
- Ni de acuerdo ni en desacuerdo
- Parcialmente en desacuerdo
- Completamente en desacuerdo

4.4 ¿Desde qué practicas deporte tienes mayor número de amigos (as)?

- Completamente de acuerdo
- Parcialmente de acuerdo
- Ni de acuerdo ni en desacuerdo
- Parcialmente en desacuerdo
- Completamente en desacuerdo

4.5 ¿Sientes que desde que te involucraste en el deporte has logrado superar de mejor manera los retos de la vida académica y personal?

- Completamente de acuerdo
- Parcialmente de acuerdo
- Ni de acuerdo ni en desacuerdo
- Parcialmente en desacuerdo
- Completamente en desacuerdo

4.6 ¿Cuál de las siguientes opciones ha sido el apoyo más importante para llegar hasta este nivel de entrenamiento?

Por favor califique de 1 a 5 todas las opciones de respuesta en donde 1 es el valor mínimo de influencia y 5 el máximo valor.

| 1. Familia | 1 | 2 | 3 | 4 | 5 |
| --- | --- | --- | --- | --- | --- |
| 1. Colegio | 1 | 2 | 3 | 4 | 5 |
| 1. Entrenador | 1 | 2 | 3 | 4 | 5 |
| 1. Amigos del barrio | 1 | 2 | 3 | 4 | 5 |
| 1. Compañeros de entrenamiento | 1 | 2 | 3 | 4 | 5 |
| 1. Funcionarios del IDRD | 1 | 2 | 3 | 4 | 5 |
| 1. Pareja | 1 | 2 | 3 | 4 | 5 |

4.7 Los amigos (as) con los (las) que compartes la mayor parte del tiempo que no entrenan son:

- Del barrio
- Otros compañeros del equipo
- De otra parte
- Del colegio - no deportistas
- No tengo muchos amigos

4.8 Consideras que tu participación en el deporte te ayuda a:

| 1. Fortalecer tu autoestima | 1 | 2 | 3 | 4 | 5 |
| --- | --- | --- | --- | --- | --- |
| 1. Mejorar la comunicación con tus amigos | 1 | 2 | 3 | 4 | 5 |
| 1. Descargar el estrés físico y emocional | 1 | 2 | 3 | 4 | 5 |
| 1. Poner tu cuerpo en forma | 1 | 2 | 3 | 4 | 5 |
| 1. Conocer a otras personas | 1 | 2 | 3 | 4 | 5 |
| 1. Alejarte de los vicios | 1 | 2 | 3 | 4 | 5 |

4.9 ¿Consideras que a futuro cuando finalice tu actividad deportiva seguirás en contacto con tus compañeros actuales?

- Completamente de acuerdo
- Parcialmente de acuerdo
- Ni de acuerdo ni en desacuerdo
- Parcialmente en desacuerdo
- Completamente en desacuerdo

4.10 ¿Consideras que podrías tener como pareja afectiva a una persona con alguna discapacidad?

- Completamente de acuerdo
- Parcialmente de acuerdo
- Ni de acuerdo ni en desacuerdo
- Parcialmente en desacuerdo
- Completamente en desacuerdo

1. **Régimen de vida**

5.1 ¿Consideras que la actividad física y el deporte te ayudan a evitar el consumo de licor, tabaco y drogas?

- Completamente de acuerdo
- Parcialmente de acuerdo
- No existe relación
- Parcialmente en desacuerdo
- Totalmente en desacuerdo

5.2 La gente que conoces y que practica deporte no consume licor, tabaco ni drogas

- Completamente de acuerdo
- Parcialmente de acuerdo
- Ni de acuerdo ni en desacuerdo
- Parcialmente en desacuerdo
- Totalmente en desacuerdo

5.3 ¿Consideras que desde que estás involucrado (a) en el deporte?

- Mejoró tu desempeño académico
- No has notado cambios significativos en tu desempeño académico
- Ha empeorado tu desempeño académico

5.4 ¿Conoces muy bien los beneficios que le generan la actividad física y el deporte?

- Completamente de acuerdo
- Parcialmente de acuerdo
- Ni de acuerdo ni en desacuerdo
- Parcialmente en desacuerdo
- Totalmente en desacuerdo

5.5 ¿Consideras que tu participación en el deporte te ha ayudado a afrontar de mejor manera los retos de otras áreas de la vida?

- Completamente de acuerdo
- Parcialmente de acuerdo
- Ni de acuerdo ni en desacuerdo
- Parcialmente en desacuerdo
- Totalmente en desacuerdo

5.6 Consideras que la participación de personas con discapacidad en el deporte ayuda a:

Por favor califique de 1 a 5 todas las opciones de respuesta en donde 1 es el valor mínimo de influencia y 5 el máximo valor.

| 1. Alejarse de vicios | 1 | 2 | 3 | 4 | 5 |
| --- | --- | --- | --- | --- | --- |
| 1. Bienestar psicológico | 1 | 2 | 3 | 4 | 5 |
| 1. Bienestar físico | 1 | 2 | 3 | 4 | 5 |
| 1. Reconocimiento social | 1 | 2 | 3 | 4 | 5 |
| 1. Fortalecer la autoestima | 1 | 2 | 3 | 4 | 5 |
|  | 1 | 2 | 3 | 4 | 5 |

5.7 Consideras que desde que practicas deporte:

|  | SI | NO | NR |
| --- | --- | --- | --- |
| 1. Tengo más amigos |  |  |  |
| 1. Ha mejorado mi autoestima |  |  |  |
| 1. Me relaciono mejor con la gente |  |  |  |
| 1. En mi familia….. |  |  |  |
|  |  |  |  |
|  |  |  |  |

**6. Confianza y autopercepción**

- 1. Me siento muy bien con mi apariencia física
- Completamente de acuerdo
- Parcialmente de acuerdo
- Ni de acuerdo ni en desacuerdo
- Parcialmente en desacuerdo
- Totalmente en desacuerdo
  1. Mi discapacidad no me impide desempeñarme bien en el deporte.
- Completamente de acuerdo
- Parcialmente de acuerdo
- Ni de acuerdo ni en desacuerdo
- Parcialmente en desacuerdo
- Totalmente en desacuerdo
  1. Considero que en el futuro tendré una buena vida.
- Completamente de acuerdo
- Parcialmente de acuerdo
- Ni de acuerdo ni en desacuerdo
- Parcialmente en desacuerdo
- Totalmente en desacuerdo
  1. Mi discapacidad no me impide desempeñarme bien en los diferentes campos de la vida.
- Completamente de acuerdo
- Parcialmente de acuerdo
- Ni de acuerdo ni en desacuerdo
- Parcialmente en desacuerdo
- Totalmente en desacuerdo
  1. Me molesta que la gente en la calle note mi discapacidad
- Completamente de acuerdo
- Parcialmente de acuerdo
- Ni de acuerdo ni en desacuerdo
- Parcialmente en desacuerdo
- Totalmente en desacuerdo

6.6 ¿Qué es lo que más te gusta de tu experiencia en la práctica de tu deporte?

1. **Relación con el entrenador**
   1. El entrenador cree en mi capacidad para lograr resultados importantes.

- Completamente de acuerdo
- Parcialmente de acuerdo
- Ni de acuerdo ni en desacuerdo
- Parcialmente en desacuerdo
- Totalmente en desacuerdo
  1. El entrenador se preocupa por mi comportamiento dentro y fuera de los espacios de entrenamientos
- Completamente de acuerdo
- Parcialmente de acuerdo
- Ni de acuerdo ni en desacuerdo
- Parcialmente en desacuerdo
- Totalmente en desacuerdo
  1. El entrenador me alienta para que yo logre los objetivos fijados
- Completamente de acuerdo
- Parcialmente de acuerdo
- Ni de acuerdo ni en desacuerdo
- Parcialmente en desacuerdo
- Totalmente en desacuerdo
  1. ¿El entrenador me da un trato condescendiente y lastimero porque tengo discapacidad?
- Completamente de acuerdo
- Parcialmente de acuerdo
- Ni de acuerdo ni en desacuerdo
- Parcialmente en desacuerdo
- Totalmente en desacuerdo

7.6 El entrenador me ayuda a establecer los objetivos de mi plan de entrenamiento anual.

- Completamente de acuerdo
- Parcialmente de acuerdo
- Ni de acuerdo ni en desacuerdo
- Parcialmente en desacuerdo
- Totalmente en desacuerdo

8. **Desafíos y aspiraciones**8.1 ¿Cuáles son los principales desafíos que enfrenta al practicar deporte? (Marque todos los que apliquen)

Por favor califique de 1 a 5 todas las opciones de respuesta en donde 1 es el valor mínimo de influencia y 5 el máximo valor.

| 1. Accesibilidad de instalaciones | 1 | 2 | 3 | 4 | 5 |
| --- | --- | --- | --- | --- | --- |
| 1. Falta de apoyo técnico | 1 | 2 | 3 | 4 | 5 |
| 1. Costos económicos | 1 | 2 | 3 | 4 | 5 |
| 1. Prejuicios o discriminación | 1 | 2 | 3 | 4 | 5 |
| 1. Otros (especificar) | 1 | 2 | 3 | 4 | 5 |

- 1. Cuáles son sus metas futuras en el ámbito deportivo?
  2. Qué cree que se podría hacer a nivel comunitario o gubernamental para fomentar la participación de personas con discapacidad en el deporte?
  3. Hay algo más que le gustaría añadir sobre su experiencia en el deporte como persona con discapacidad?

9.Contacto del participante:

Nombre: ________________________________

Correo electrónico: ________________________

Teléfono: ________________________________
