# Supplementary material for: Determinants of participation and support mechanisms in a paralympic sport training program: evidence from the IDRD program in Bogotá
Source: Front Sports Act Living. 2026 Jul 3;8:1882854. doi: 10.3389/fspor.2026.1882854 (PMC13377818; doi:10.3389/fspor.2026.1882854)
Supplement: Supplementary file 2 [file Supplementaryfile2.docx]

**APPENDIX B – SUPPLEMENTARY RESULTS**

*Note.* Vertical axis represents scores on a 5-point Likert scale ranging from 1 (“strongly disagree”) to 5 (“strongly agree”).

Figure S1. *Average scores by type of barriers to participation in sport training programs.*
